# Transcutaneous Vagus Nerve Stimulation Induces Tidal Melatonin Secretion and Has an Antidiabetic Effect in Zucker Fatty Rats

**DOI:** 10.1371/journal.pone.0124195

**Published:** 2015-04-16

**Authors:** Shuxing Wang, Xu Zhai, Shaoyuan Li, Michael F. McCabe, Xing Wang, Peijing Rong

**Affiliations:** 1 Department of Anatomy, Xinxiang Medical University, Xinxiang, Henan Province, China; 2 Department of Physiology, Institute of Acupuncture and Moxibustion, China Academy of Chinese Medical Sciences, Beijing, China; 3 MGH Center for Translational Pain Research, Department of Anesthesia, Critical Care, and Pain Medicine, Massachusetts General Hospital, Harvard Medical School, Boston, Massachusetts, United States of America; 4 Guangdong Landau Biotechnology Inc. Ltd., Guangzhou, Guangdong, China; Baylor College of Medicine, UNITED STATES

## Abstract

Melatonin plays a protective role in type 2 diabetes (T2D) through regulation of glucose metabolism. Whether transcutaneous vagus nerve stimulation (taVNS) is antidiabetic and whether a modulated melatonin production is involved in the antidiabetic mechanism of taVNS is unknown. In this study, once daily 30min noninvasive taVNS was administered in Zucker diabetic fatty (ZDF, fa/fa) and Zucker lean (ZL, +/fa) littermates under anesthesia for 5 consecutive weeks. The acute and chronic influences of taVNS on the secretion of melatonin were studied as well as the effects of taVNS on blood glucose metabolism. We found that naïve ZDF rats develop hyperglycemia naturally with age. Each taVNS session would trigger a tidal secretion of melatonin both during and after the taVNS procedure and induce an acute two-phase glycemic change, a steep increase followed by a gradual decrease. Once daily taVNS sessions eventually reduced the glucose concentration to a normal level in seven days and effectively maintained the normal glycemic and plasma glycosylated hemoglobin (HbA_lc_) levels when applied for five consecutive weeks. These beneficial effects of taVNS also exist in pinealectomized rats, which otherwise would show overt and continuous hyperglycemia, hyperinsulinemia, and high HbA_lc_ levels. We concluded that multiple taVNS sessions are antidiabetic in T2D through triggering of tidal secretion of melatonin. This finding may have potential importance in developing new approaches to the treatment of T2D, which is highly prevalent, incurable with any current approaches, and very costly to the world.

## Introduction

Type 2 diabetes (T2D) is a metabolic disorder characterized by high blood sugar levels due to increased insulin resistance, in which the body’s cells have lost the ability to respond adequately to relatively normal levels of insulin [1]. T2D is highly prevalent in adults but incurable with any of the current therapeutic approaches [2]. Physiologically, the metabolism of blood glucose is mainly and directly regulated by glucagon secreted from pancreatic α-cells in response to low glucose levels and insulin from β-cells to high glucose levels. Additionally, insulin secretion is regulated by growth hormone, glucagon like peptide-1, and other hormones and substances [1].

Melatonin (N-acetyl-5-methoxytryptamine) is the hormone that functions as the mediator of photoperiodic information to the central nervous system in vertebrates and allows central circadian regulation of numerous physiological homeostatic mechanisms [3–6]. It was recently discovered that melatonin plays a protective role against T2D through regulation of glucose metabolism in animals [3,5] and patients [4] via changes in insulin secretion and leptin production [2].

Acupuncture has been shown to decrease blood glucose level in T2D [7] and is beneficial in the treatment of obesity [8], which is a primary cause of T2D in genetically predisposed people [2]. There are vagal innervations in the auricular concha region [9]. The autonomic nervous system, including the vagus nerve, plays a critical role in the neuroendocrine homeostasis [10]. Transcutaneous electronic stimulation at the auricular region has been shown to be beneficial to depression patients and rodents, and it is believed that the stimulation works through the vagus nerve [11–17].

In our previous studies, taVNS ameliorated depression [9], a condition characterized by low circulating melatonin. Considering that a circadian rhythm of melatonin is necessary in maintaining insulin sensitivity [18] and that the rhythm may be lost in ZDF rats [3,4], especially in pinealectomized ZDF rats [19], restoring rhythmic melatonin secretion will be helpful in treating T2D. In this project, using the genetically diabetic ZDF rats as T2D model and ZL rats as control, we studied whether taVNS would be antidiabetic through modulation of melatonin production.

## Methods

### Diabetic animal model

Male Zucker diabetic fatty (ZDF, fa/fa) rats and Zucker lean (ZL,+/fa) littermates were purchased at 5wk old from Vital River Laboratories International Inc. (Beijing, China). Animals were housed under controlled temperature (21°C±2°C), relative humidity (50%±10%) and artificial light (12 h light/dark cycle, lights on at 7 A.M.). Littermates from the same or foster mother were housed in one large cage with distilled water and a standard rat diet pellets available *ad libitum* except on the days when the rats be fasted for 6 hours and a blood glucose level test will be taken. The body weight was recorded weekly and average daily food intake calculated in a whole for each group during the experiment. Rats entered the experimental procedure at 8wk of age, divided into ZDF and ZL groups according to the rat’s size, appearance, and blood glucose level. The ZDF or ZL rats were further divided into subgroups randomly. The investigators were not blinded to the group allocation during the experiment because treatment procedures, for example the taVNS or melatonin injection, must be kept in one group, and the tests used in this study are all objective. For this study, only male ZDF rats of the same age were used to avoid a possible confounding effect from gender and age differences on the levels of endogenous melatonin, glucagon, insulin, and other possible hormones. The experimental protocol was approved by the Institutional Animal Care and Use Committee in China Academy of Chinese Medical Sciences. Principles of laboratory animal care were followed.

### Noninvasive transcutaneous auricular vagus nerve stimulation

**Fig 1:**
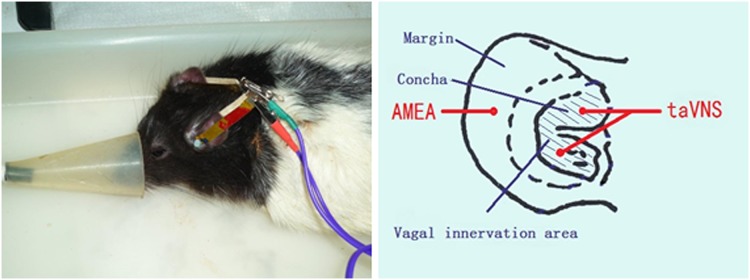
The electroacupuncture procedure. Showing the auricular stimulation posture (a) and location (b) of the procedure. AMEA: stimulation applied at auricular margin; taVNS: stimulation applied at auricular concha.

All the time points recorded in this study are in accordance with the taVNS occurrences, i.e. the first taVNS session occurs on day 1, the seventh day recorded as W1, and so on. For taVNS, under 2% isoflurane inhalation anesthesia, two oppositely charged magnetic electrodes (+/-) were placed over the auricular concha region, inside and outside respectively, of each ear. Saline was applied between an electrode and the skin to improve electric conductivity. A session of 30min transcutaneous electrical stimulation at frequencies of 2/15 Hz (2 and 15 Hz, switched every second) and an intensity of 2mA was applied via an electrical stimulator (HANS-100, Nanjing, China). The procedures were given in the afternoon after a blood glucose concentration test and a blood sample collection at designated time points. Auricular margin was used as the sham acupoint. The electroacupuncture condition at auricular margin was same as that at taVNS except the stimulate location (Fig 1).

### Blood glucose concentration testing

By using Ascensia Breeze Blood Glucose Monitoring System (Newbury, Berks, UK), the glucose concentration was tested from tail tip-prick blood samples. To determine the acute effects of taVNS on glucose concentration, blood glucose was tested for the first 5 consecutive days, 6 times per day: immediately before, 15min during, immediately after, 30, 60, and 120 min after the taVNS procedure. The chronic effect of taVNS on glucose metabolism was determined by weekly measurements of glucose concentration at 0 (baseline), 1, 2, 3, 4, and 5 weeks, before daily taVNS or melatonin treatments. The test range was 0.6–33.3 mmol/L. Any concentration over the testing limit was recorded as 33.3 mmol/L for statistical purpose.

### Melatonin injection

For the melatonin injection group, melatonin was injected intraperitoneally once daily in the afternoon and after blood glucose test (4 P.M.) at designated time points. Melatonin was purchased from Sigma Chemical Co. (St. Louis, USA) and dissolved in 5% ethanol saline (v/v) immediately before use. The injected dose was 60mg/kg body weight.

### Pinealectomy

To find out the role of taVNS in pinealectomized rats and to explore whether there are other melatonin sources, the blood concentration of glucose, melatonin, insulin, and HbA_lc_ were examined in pinealectomized rats. The pineal gland was removed from rats according to a reported method [20]. Briefly, male ZDF rats were intraperitoneally anesthetized with 50 mg/kg sodium pentobarbital. The animal was fastened to a stereotaxic apparatus; an incision along the skull middle line was made to expose the lambda. The skull at the lambda was opened with a dental drill with a circle bit (0.5 cm Outside Diameter) so that the superior sagittal sinus was directly under view. The superior sagittal sinus was ligated with 5–0 silk suture at the rostral side for two ligations, cut between, and carefully pulled back. The pineal gland, located under the venous sinus, was removed in a single piece using tweezers and the bone fragment was returned to its place, the surgical layers were sutured. Sham pinealectomy was operated the same way but leave the pineal gland intact in place. Rats exhibiting neurological deficits (e.g., paralysis) or postoperative poor grooming were euthanatized by intraperitoneal injection of sodium pentobarbital (200 mg/kg) and excluded from further experiments. Two weeks after the pinealectomy procedure, the rats were subjected to consecutive taVNS and the blood concentration of glucose and the hormones were tested at designated time points.

### Collection of plasma and ELISA

Blood samples were collected from right atrium upon final sample collection or from tail tip veins at other time points, centrifuged for 10min at 110 g in cold room and plasma was collected. All plasma samples were wrapped in foil and stored at -80°C until use. Concentrations of plasma melatonin, serotonin, glucagon, insulin, and HbA_lc_ were evaluated using enzyme linked immunosorbent assay (ELISA) kits purchased from R&D System (Beijing, China) and analyzed by Huanya Biomedicine Technology Co. LTD (Beijing, China). The results were read using a microplate reader (Multiskan MK3, Thermo Scientific, Beijing, China) at wavelengths of 450nm.

### Statistical analysis

By running GraphPad InStat version 3 for Windows, raw data of body weight, food intake, glucose concentration, and ELISA results at last time point were compared using repeated measure One-way ANOVA to detect differences among treatment groups, followed by Tukey-Kramer Multiple Comparisons Test to determine sources of differences. Data were presented as mean ± standard deviation. Differences were considered to be statistically significant at the level of α = 0.05. For the acute effect of taVNS on the secretion of hormones during taVNS session and two hours immediately after it, only average of raw data and standard difference were used to produce the dotted lines with error bar for secretion-shape and secretion-duration comparing propose.

## Results

### Effects of different treatments on body weight and food intake of rats

The body weight change in naïve rats would be considered natural. Compared with that in naïve rats, the body weight of taVNS treated ZL rats was lower after three weeks of treatment and for ZDF rats two weeks after. For the daily melatonin administrated ZDF rats, the body weight was comparably lower than naïve ZDF rats two weeks after the beginning of injection (Table 1).

**Table 1:** The weekly body weight of rats in grams.

Wk	Naive		AMEA		taVNS		Melatonin
	ZL	ZDF	ZL	ZDF	ZL	ZDF	ZDF
0	283.8±5.3	311.3±4.7	283.8±4.9	310.3±5.2	285.5±4.3	313.7±5.1	312.8±4.9
1	289±5.6	338.5±6.5	287.5±6.4	329.3±4.3	292±5.9	339.8±6.5	325.8±6.9
2	313.3±6.9	374.5±6.9	314.3±6.3	367±7.5	302.5±8.1	360.7±6.9	350.5±9.1*
3	320.3±7.5	393.8±7.9	327±7.7	390.8±7.9	304.3±8.9	371.3±9.1*	365±9.8*
4	333.8±8.2	412.3±9.6	335.8±8.3	404±9.5	311.3±9.7*	383.3±11**	379.1±10.9**
5	348.8±8.7	415.5±11.3	338.3±9.3	408.5±12.7	310±9.7**	386±12.9**	384.2±11.3**

Each value represents mean ± SD (n = 6 each group).

**, P<0.01,

*, P<0.05 versus naïve rats of the same genotype and at the same time point.

As compared for the whole duration of treatments, the average daily food intake for different treatment groups was not significantly different except the melatonin injection ZDF rats, which ate less food (P<0.05) as compared with naïve ZDF rats (Table 2).

**Table 2:** Average daily food intake (grams) of rats during the experiment.

Naive		AMEA		taVNS		Melatonin
ZL	ZDF	ZL	ZDF	ZL	ZDF	ZDF
19.2±3.2	28±2.9	17.5±2.4	27.3±3.1	17.4±2.7	29.1±3.6	24.2±3.2*

Each value represents mean ± SD (n = 6).

*P<0.05 as compared with naïve ZDF rats.

### Acute effects of taVNS on plasma concentrations of melatonin, glucagon, and insulin of ZDF rats

**Fig 2:**
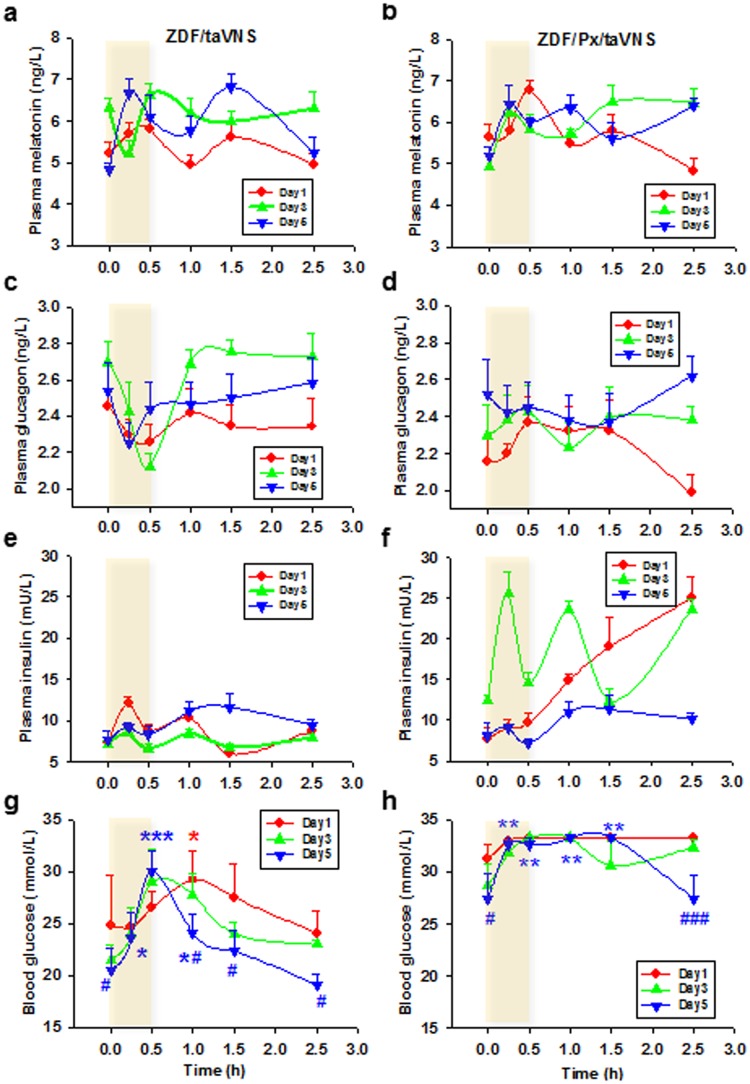
Acute effects of taVNS in ZDF rats. Comparison between naïve ZDF rats (left column, n = 5) and pinealectomized ZDF rats (two weeks after pinealectomy operation) (right column, n = 5), as well as among day 1, 3, and 5 of the consecutive once-daily-30min-taVNS treatment (shadow area), changes in plasma concentration of melatonin (a, b), glucagon (c, d), and insulin (e, f), and blood glucose levels (g, h). 0.0–3.0, elapsed time in hour beginning from the start of the taVNS treatment. *, **, *** P<0.05, 0.01, 0.001 vs. baseline (0.0) of the same day; #, ### P<0.05, 0.001 vs. day 1 at the same abscissa point, respectively. Sample size estimated based on power level of 0.8.

As detected from samples taken on day 1, 3, and 5, each taVNS session would induce tidal melatonin release with multiple parabolic waves. The waves existed both during and after the taVNS session (Fig 2a). A comparison between the during- and after-taVNS waves shows the latter as having a longer period and lower frequency, while retaining amplitude. As compared between days, the melatonin waves tend to be higher or more frequent as the rats participated in more sessions (Fig 2a and 2b). The taVNS triggered acute, tidal, and rhythmic melatonin secretions existed in intact (Fig 2a) as well as in pinealectomized ZDF rats (Fig 2b), indicating that taVNS triggers extrapineal melatonin secretion.

Both of the concentrations of glucagon (Fig 2c and 2d) and insulin (Fig 2e and 2f) also changed in tidal manner. However, neither glucagon nor insulin concentrations changed in exact rhythm with the release of melatonin (Fig 2a and 2b).

### Effects of taVNS on blood glucose concentrations in ZDF rats

**Fig 3:**
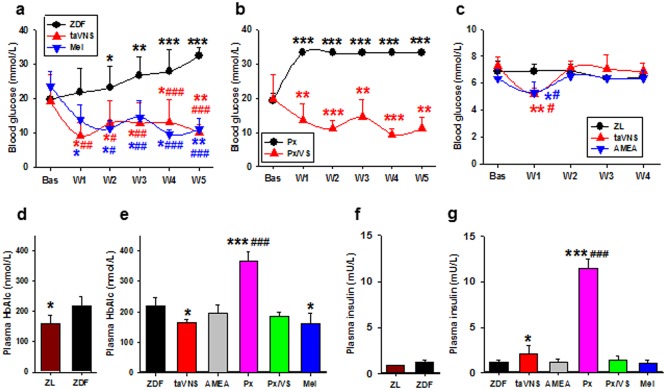
Chronic effects of taVNS in rats. Comparing blood glucose concentrations in naïve and taVNS treated ZDF rats (a, n = 6 each), in pinealectomized ZDF rats (immediately after pinealectomy operation) with or without taVNS treatment (b, n = 5 each), and in naïve, taVNS, and AMEA treated ZL rats (c, n = 4 each). ZL, ZDF, naïve ZL or ZDF rats; taVNS, taVNS treated rats; AMEA, auricular margin electroacupuncture treated rats; Px, pinealectomized rats; Px/VS, taVNS treated pinealectomized rats; Mel, daily melatonin injected rats. Bas, baseline (before taVNS); W1–W5, 1–5 weeks after consecutive taVNS treatment. *, **, *** P<0.05, 0.01, 0.001 *vs*. Bas of the same group; #,##,### P<0.05, 0.01, 0.001 *vs*. naïve at the same time point, respectively. Concentrations of plasma HbA_1c_ (d, e) and insulin (f, g) upon sampling were compared in naïve ZDF and ZL rats (d, f) and in ZDF rats subjected to different treatments (e, g). *, *** P<0.05, 0.001 *vs*. ZDF, respectively; ### P<0.001 *vs*. remaining groups. Sample size estimated based on power level of 0.8.

At 8 weeks of age, the glycemic level in naive ZDF rats was 19.793±7.158 mmol/L (mean ± SD, n = 14; 95% CL, 15.661–23.925). ZDF rats develop hyperglycemia naturally with age but the procession could be broken/reversed by taVNS (Fig 3a). Although each taVNS session produced immediate multiple, fluctuating waves of melatonin, glucagon, and insulin, the wave for blood glucose level shifted downward day by day, such that the glucose concentration was significantly lower on day 5 than that on day 1, both before and 2h after taVNS session, and in both intact and pinealectomized ZDF rats (Fig 2g and 2h).

The antidiabetic effects of long term taVNS treatment is reflected on the well-controlled glucose metabolism in taVNS treated ZDF rats, as compared with that in naïve (Fig 3a) and pinealectomized animals without taVNS or complimentary melatonin (Fig 3b). After seven consecutive taVNS treatments, glucose reached low levels and was well maintained by daily taVNS treatment for the entire experiment duration (Fig 3a and 3b). In pinealectomized ZDF rats not treated with taVNS or complementary melatonin, hyperglycemia existed at all tested time points after surgery (Fig 3b) and an overt hyperinsulinemia was presented at time of sampling (Fig 3f and 3g).

### Effects of taVNS on blood glucose concentrations in ZL rats

The baseline glycemic concentration of ZL rats was 6.733±0.724 mmol/L (mean ± SD; n = 12; 95%CL, 6.273–7.193). It decreased to a lower level at week one of the consecutive daily electric stimulation in both taVNS and auricular margin electroacupuncture (AMEA) treated rats. The taVNS showed no significant influence on the glycemic level in ZL rats thereafter (Fig 3c).

### Effects of taVNS on plasma concentrations of HbA_lc_

At an age of 12 wks, the plasma HbA_lc_ in naive ZDF rats was significantly higher than in ZL littermates (Fig 3d). While pinealectomy in ZDF rats would elevate HbA_lc_ to a much higher level, taVNS or melatonin treatment maintained it at a lower level comparable with that in naive ZL rats (Fig 3e).

### Plasma melatonin and serotonin concentrations at last time point in rats with different treatments

The effect of long-term treatments on the secretion of melatonin, including taVNS, AMEA, as well as melatonin injection, was shown in Table 3. Interestingly, only the melatonin secretion in taVNS treated ZDF group was significantly increased (Table 3). In contrast, there was no statistical differences of serotonin (5-HT) concentration between any two groups (Table 4).

**Table 3:** Plasma melatonin concentration (ng/L) in rats at last time point.

Naive		AMEA		taVNS		Melatonin
ZL	ZDF	ZL	ZDF	ZL	ZDF	ZDF
5.4±0.31	3.7±0.46	5.6±0.48	3.9±0.39	5.9±0.52	4.7±0.31* ^#^	3.4±0.54

Each value represents mean ± SD (n = 6).

*, P<0.05, versus N-ZL;

#, P<0.05 versus N-ZDF.

**Table 4:** Plasma 5-HT concentration (ng/L) in rats at last time point.

Naive		AMEA		taVNS		Melatonin
ZL	ZDF	ZL	ZDF	ZL	ZDF	ZDF
8.55±3.16	8.66±.92	6.92±3.51	15.34±6.14	19.53±12.85*	15.91±10.48^#^	10.96±5.59

Each value represents mean ± SD (n = 6).

*, P<0.05, versus N-ZL;

#, P<0.05 versus N-ZDF.

### Effect of long term melatonin administration in ZDF rats

The group of ZDF rats treated with once daily melatonin injection was used as a positive control. Results from this group were distributed in Fig 3 and tables 1,2, 3, and 4. In general, melatonin showed an effective antidiabetic efficacy in ZDF rats, including lower body weight (Table 1), decreased blood glucose concentration (Fig 3a), and repressed insulin concentration (Fig 3g). However, melatonin reduced the food intake (Table 2). The melatonin concentration at last time point in the melatonin injected rats was not changed (Table 3).

## Discussion

In this study we found that taVNS triggers melatonin release in a tidal manner and inhibits the progression of T2D. Physiologically, melatonin is secreted from both pineal and extrapineal sources [21–26]. The pineal melatonin is secreted in the night while the extrapineal melatonin secretion, as induced by taVNS, may be different from pineal melatonin secretion. Since the production of melatonin from pineal gland in T2D may be defective [3,4], some of the taVNS triggered melatonin production in intact pineal ZDF rats and in pinealectomized rats may be extrapineal, which normally accounts for only 20% of circulating melatonin [23]. Besides the pineal gland, melatonin is released from the digestive tract, bone marrow, and many other sources [23–25]. These extrapineal sources have a huge total volume and some are quite rich in melatonin, for example, melatonin concentrations in both the digestive tract and bone marrow surpass that in blood by 2 to 3 orders of magnitude and the digestive tract alone releases over 400x more melatonin than from pineal gland [24,25]. Extrapineal melatonin secretion may be out of light signal control. It is known that the digestive tract releases melatonin upon rhythmic food ingestion from enterochromaffin cells, which contain precursors of melatonin and complimentarily increase in numbers after pinealectomy [26]. Possibly that’s the reason why the melatonin concentration around taVNS cessions is not significantly different between the pineal intact and pinealectomized rats. However, we can’t reach the conclusion that extrapineal sources would compensate the pineal melatonin secretion yet. Whatever the source, melatonin shows the definitely ability to influence the body weight and also possibly food intake.

Melatonin modulates glucose metabolism through receptor-dependent influences on glucagon and insulin secretion [19,27,28]. In pancreatic islets, the melatonin receptor type 1 (MT1) is expressed on α-cells while the type 2 (MT2) on β-cells [19,28].

It has been reported that melatonin increases glucagon production from pancreatic α-cells [27] but inhibits insulin production in β-cells [29]. In the current study, although both of the concentrations of glucagon and insulin changed in tidal manner related to the tidal concentration change of melatonin, neither glucagon nor insulin concentrations changed in exact rhythm with the release of melatonin. These results may indicate that melatonin modulates the secretion of both glucagon and insulin, and that possibly due to the different half-lives of the three hormones in blood, different wave lengths exhibited.

Melatonin is reported to inhibit insulin production through an MT2 receptor mediated cGMP signaling pathway in β-cells [29] with a functional phase-shift following the binding of melatonin to its receptor [6]. In T2D rats, insulin secretion may lose part of this negative regulatory mechanism resulting in hyperinsulinemia [3,4], especially in pinealectomized ZDF rats in this study. Two weeks after the pinealectomy procedure, although the β-cells in these rats could start insulin secretion upon each taVNS treatment, the secretion seemingly was the by-product of melatonin negative control upon the first taVNS session such that significantly high insulin levels were steadily reached. Even upon the third taVNS session, the insulin concentration still changed wildly. However, upon the fifth taVNS session, insulin concentration during and following taVNS session in the same pinealectomized ZDF rats showed a tidal manner as that in pineal intact ZDF rats. These results indicate that, i) the production of insulin in β-cells of T2D may exist, ii) a rhythmic secretion of melatonin is important to maintain the regulatory function of insulin release, and iii) the consecutive daily taVNS sessions can eventually restore the tidal secretion of melatonin and hold the control of insulin secretion.

The α- and β-cells may not release hormones at the same rhythm for reasons that, acute exposure of MT2 to physiological or supraphysiological concentrations of melatonin would induce a concentration- and time-dependent receptor desensitization and internalization, which would take 8–24 h to resensitize [30], whereas prolonged exposure to a melatonin concentration mimicking nocturnal levels did not affect the number of MT1, their affinity or functional sensitivity [31]. Instead, α-cell-activation may have a prerequisite role in β-cell-activation: (i) without the MT1 involved, melatonin treatment would not show significant effects on insulin release [30]; and (ii) a number of single nucleotide polymorphisms in the MTNR1B gene that encode for MT2 are involved in the pathogenesis of T2D [32], however, loss-of-function mutations in or removal of MT1 would significantly impair the ability to metabolize glucose [33].

Although taVNS is antidiabetic in ZDF rats, it has limited efficiency in ZL control. This may be due to a hyperinsulinemia induced sympathetic hyperactivity which reduces melatonin production and increases insulin resistance in T2D rats [21]. The taVNS session has shown the capability to restore the autonomic balance [9,22]. However, further studies are needed to find out whether hyperglycemia would recur following the discontinuation of taVNS treatment in T2D.

**Fig 4:**
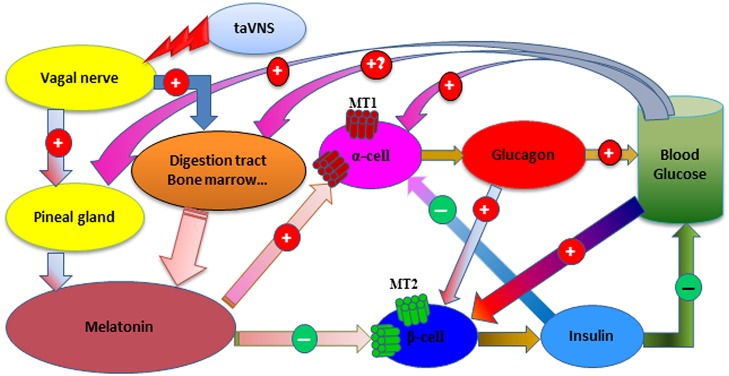
Schematic depiction of the modulation of glucose metabolism following taVNS in T2D. See Discussion for interpretation.

A theoretic cascade in T2D following taVNS is shown in Fig 4. Briefly, (i) taVNS stimulates the auricular vagal branches, increases the parasympathetic tone, and triggers initial melatonin releases; (ii) melatonin activates α-cells to release glucagon and thus elevates glycemic level; (iii) the released glucagon and the elevated glucose level trigger an instant insulin release from β-cells to reduce the glycemic level and simultaneously inhibit glucagon release; meanwhile, melatonin combines with MT2 receptors on β-cells and inhibits insulin production, thus a delayed but sustained insulin release follows the initial surge; (iv) at a low enough point, the glucose concentration activates epinephrine secretion to trigger melatonin production [34] thus another circle begins. Considering the half-lives of around 10–15 min for insulin [35] and 20 min for melatonin [36], the delayed reaction time of β-cells upon MT2 activation, and the response time of blood glucose, each cycle may take hours.

Our current findings may have potential importance in developing new approaches to the treatment of T2D, which is highly prevalent, incurable with any current approaches, and very costly to the world. We suggest taVNS or short half-life melatonin agonists for the treatment of the illness. One of the advantages of our finding is that taVNS clinics can be easily setup worldwide.
